# The AIP Model of EMDR Therapy and Pathogenic Memories

**DOI:** 10.3389/fpsyg.2017.01578

**Published:** 2017-09-21

**Authors:** Michael Hase, Ute M. Balmaceda, Luca Ostacoli, Peter Liebermann, Arne Hofmann

**Affiliations:** ^1^Lüneburger Zentrum für Stressmedizin Lüneburg, Germany; ^2^Therapie Lüneburg Lüneburg, Germany; ^3^School of Medicine, University of Turin, San Luigi Gonzaga University Hospital Turin, Italy; ^4^Private Practice for Psychiatry and Psychotherapy Leverkusen, Germany; ^5^EMDR-Institute Deutschland Bergisch Gladbach, Germany

**Keywords:** EMDR therapy, mental disorders, pathogenic memory, psychotherapy, PTSD, psychosomatic medicine

## Abstract

Eye Movement Desensitization and Reprocessing (EMDR) therapy has been widely recognized as an efficacious treatment for post-traumatic stress disorder (PTSD). In the last years more insight has been gained regarding the efficacy of EMDR therapy in a broad field of mental disorders beyond PTSD. The cornerstone of EMDR therapy is its unique model of pathogenesis and change: the adaptive information processing (AIP) model. The AIP model developed by F. Shapiro has found support and differentiation in recent studies on the importance of memories in the pathogenesis of a range of mental disorders beside PTSD. However, theoretical publications or research on the application of the AIP model are still rare. The increasing acceptance of ideas that relate the origin of many mental disorders to the formation and consolidation of implicit dysfunctional memory lead to formation of the theory of pathogenic memories. Within the theory of pathogenic memories these implicit dysfunctional memories are considered to form basis of a variety of mental disorders. The theory of pathogenic memories seems compatible to the AIP model of EMDR therapy, which offers strategies to effectively access and transmute these memories leading to amelioration or resolution of symptoms. Merging the AIP model with the theory of pathogenic memories may initiate research. In consequence, patients suffering from such memory-based disorders may be earlier diagnosed and treated more effectively.

## Introduction

Eye Movement Desensitization and Reprocessing (EMDR) therapy was introduced in 1987 as a treatment for post-traumatic stress disorder (PTSD). EMDR therapy is not only an evidence-based treatment of PTSD (Bisson and Andrew, 2007; Watts et al., 2013; World Health Organization [WHO], 2013; Schulz et al., 2015), but is also a potentially effective treatment for various other mental disorders as affective disorders (Landin-Romero et al., 2013; Hofmann et al., 2014; Novo et al., 2014; Hase et al., 2015), chronic pain (Schneider et al., 2005; Wilensky, 2006; de Roos et al., 2010; Gerhardt et al., 2016), addiction (Hase et al., 2008; Abel and O’Brien, 2010), or obsessive compulsive disorders (Marsden et al., 2017). Functional imaging studies enable us to understand the working mechanisms of EMDR therapy to a great extent (Pagani et al., 2012; Lee and Cuijpers, 2013).

F. Shapiro developed a model of pathogenesis and change based on her experiences in EMDR therapy treatment sessions. This model is unique to EMDR therapy and is called adaptive information processing (AIP) model, abbreviated AIP model (Shapiro, 2001a). Since then the development and practice of EMDR therapy has been guided by the AIP model.

One of the key tenets of the AIP model predicts that dysfunctionally stored and not fully processed memories are the cause of a number of mental disorders, including, e.g., PTSD, affective disorders, chronic pain, addiction, and various other disorders. However, the exact nature of memory and its mechanism in detail is far more difficult to determine than the fact that after a certain event, a certain psychopathology appears, which can be effectively addressed by EMDR therapy.

## The AIP Model of EMDR Therapy

From her experiences in EMDR treatment sessions, Shapiro developed a unique theoretical model for the pathogenesis and change relating to EMDR therapy (Shapiro, 2001a,b). Since then, EMDR therapy has been guided by the AIP model (Shapiro, 2007; Shapiro and Laliotis, 2011). The AIP model focuses on the patient’s resources. Within the AIP model, one assumes that the human brain can usually process stressful information to complete integration. Only if this innate information processing system is impaired, the memory will be stored in a raw, unprocessed, and maladaptive form. A particularly distressing incident may then become stored in state-specific form. This implies also the inability to connect with other memory networks that hold adaptive information. Shapiro hypothesizes that when a memory is encoded in such excitatory, state-specific form, the original perceptions can be triggered by a variety of internal and external stimuli. In the view of the AIP model dysfunctionally stored memories form the basis for future maladaptive responses, because perceptions of current situations are automatically linked with associated memory networks of these unprocessed, dysfunctionally stored memories. For instance childhood experiences also may be encoded with survival mechanisms and include feelings of danger that are inappropriate for adults. However, these past events retain their power because they have not been appropriately assimilated over time into adaptive networks (Solomon and Shapiro, 2008). One of the key tenets of the AIP model is that these dysfunctionally stored and not fully processed memories form the basis of psychopathology. Activation of these memories, even years after the event, can lead to a spectrum of symptoms including intrusions that can range from an overwhelming experience, mostly called flashback, to barely noticeable intrusions. These memories lack the feeling of remembering, as described by Barry as memories without “memory awareness” (Barry et al., 2006). This contributes to the lively, actual experience, and sometimes makes it difficult to connect symptoms to the memories behind them.

The overwhelming experience and high amount of traumatic stress in a traumatic experience according to the Diagnostic and Statistical Manual of Mental Disorders (DSM-V) (American Psychiatric Association [APA], 2013) can be assumed to explain the disruption in information processing. But there can be many more causes imaginable as clinical experiences show (Hase and Balmaceda, 2015). Intense feelings of helplessness beside traumatic events or misinterpretations of an event as being extremely dangerous could also have these consequences. Other intense emotions based in previous experiences could lead to disruption in information processing. With children and adolescents the attachment to a caregiver or a sense of meaning seems to be a prerequisite for the processing of a stressful life experience. Accordingly the absence of an attachment figure could lead to impairment in information processing and thus to the development of PTSD even in the absence of a criterion A event (Verlinden et al., 2013). Of course abusive behavior of an attachment figure or neglect would likely lead to such consquences. Exhaustion and physical conditions in somatic disorders could explain the disruption in information processing as well as the influence of drugs in drug rape or during medical procedures. Of course this short list of possible causes is not comprehensive. It needs more rigorous research to determine the prerequisites beyond type A trauma.

In accordance with the AIP model these dysfunctionally stored memories become the focus of EMDR protocols and procedures in order to activate the information processing system thus transmuting these memories by so-called “reprocessing.” The subsequent integration into adaptive memory networks leads to a resolution of symptoms and enables learning (Solomon and Shapiro, 2008).

## Pathogenic Memories

Although the scientific discourse tends to associate memories that create intrusions with criterion A events and the definition of PTSD, non-criterion A events have been shown to create even more intrusions than criterion A events (Gold et al., 2005). Additionally, data from a survey of 832 adult subjects indicated that stressful life events can generate at least as many PTSD symptoms as traumatic events (Kendler et al., 2003). McFarlane (2010) showed that stressful life experiences can lead to intrusions without a fully developed PTSD. McFarlane (2010) also demonstrated that these intrusions relate to many mental disorders and poor health in general. Following these findings intrusions seem to be a common memory-based symptom, which is not necessarily linked with a PTSD diagnosis or criterion A event. Nevertheless, intrusions indicate a memory-based pathology beyond PTSD that can be linked with other mental disorders. This is consistent with a publication of Heinz et al. (2016) discussing basic learning mechanisms as representations of a basic dimension of mental disorders. They advocate for a research focus on such basic dimensions rather than pursuing a narrow focus on single disorders.

Centonze et al. (2005) described the importance of pathogenic memories from a theoretical perspective. There approach is based on the increasing acceptance of theories that relate the origin of many psychiatric symptoms to the formation and consolidation of implicit dysfunctional memory (Centonze et al., 2005). Since their publication other prominent authors have engaged in this discussion. Alberini and LeDoux (2013) summarize research on memory reconsolidation and dwell on the therapeutic perspective. In their opinion further research on memory reconsilidation could help to ameliorate maladaptive memories and potentiate adaptive behaviors in psychopathology (Alberini and LeDoux, 2013). Sillivan et al. (2015) explore the possibilities of latest research on epigenetic modification. They advocate for a recognition of the contribution of epigenetic mechanisms to how pathological memories associated with addiction and PTSD are stored, expressed, and subsequently modified, possibly leading to novel therapeutic targets (Sillivan et al., 2015).

Summarizing current neurobiological research, Centonze et al. (2005) state: “Experimental research examining the neural bases of non-declarative memory (such as habit formation, classical conditioning, and fear conditioning) has offered intriguing insight into how functional and dysfunctional implicit learning affects the brain.” They give evidence on the importance of long-term modification of synaptic transmission in particular as the most plausible mechanisms underlying memory trace encoding compulsions, addiction, anxiety, and phobias. Compulsions and other stereotypies are viewed as pathological habits (nearly automated implicit motor abilities) encoded as aberrant synaptic plasticity in the corticobasal ganglia loop. Centonze et al. (2005) refer to addictive drugs abusing the molecular mechanisms of reward-based associative learning by inducing long-term changes in synaptic effectiveness in those brain areas serving basic biological needs, such as feeding and sexual interaction. Finally, anxiety, panic disorder, and phobias are viewed as uncontrolled and repetitive defensive reactions secondary to abnormal fear conditioning – a form of implicit associative learning, encoded as long-term potentiation (LTP) in the lateral amygdala. In consequence, Centonze et al. (2005) propose that an effective psychotherapy must be directed to erase maladaptive pathogenic memories and research should focus on the development of techniques to remove pathogenic memories. Although they mentioned neither the AIP model, nor EMDR therapy, the concept of pathogenic memories could probably open another view on recent developments in EMDR research.

It seems to be of interest to explore the overlap of the theory of pathogenic memory and the AIP model, regarding practical implications for EMDR therapy in reprocessing maladaptive implicit memories, especially as the cited authors are advocating for the developments of therapeutic tools to modify pathogenic memories. As Centonze et al. (2005) coined the term “pathogenic memory” but did not give a precise definition, one should start here.

## Definition and Perspective

A clinical core feature of a pathogenic memories would be experiencing intrusions while the memory is activated, e.g., by sensory cues. A second feature of such memories may include vegetative arousal or other biological activity. Vegetative arousal may be felt by the patient when the memory is activated. EMDR therapists use this arousal to measure the “subjective level of disturbance” (also called SUD = subjective units of disturbance) in EMDR therapy. Craving and pain can be also understood as intrusions and assessed in similar ways (subjective level of urge, subjective level of pain). Studies show that if the memory is reprocessed in EMDR therapy, the vegetative arousal linked to the memory subsides and the SUD scores indicate change or, e.g., pain is reduced.

In addition the definition of trauma could loose some significance. The future question would not be about how traumatic an event is, but rather on the pathology developing after the event. This could lead to better understanding of the processing of certain “non-traumatic,” but nevertheless pathogenic memories within EMDR therapy. Considering the experiences of EMDR clinicians worldwide, the number of patients suffering from pathogenic memories may be much greater than that of patients suffering from PTSD alone.

Patients who may benefit from this conceptual expansion of memory pathology and subsequent reprocessing with EMDR could be suffering from a variety of mental disorders as laid out in the section “Introduction.” We will now focus on addiction, pain, and affective disorders as there seems to be more background by research or evidence by controlled studies.

(A) Patients with addiction disorders. A specific “addiction memory” was already postulated by Wolffgramm in 1995 from his studies of animal models (Wolffgramm and Heyne, 1995; Heyne et al., 1999). Wolffgramm and Heyne (1995) postulated that addiction memory contributes to craving and the chronic course of addiction. Interestingly, the removal of the addiction memory by altering the brain’s ability to learn led to a complete remission of the disorder, at least in Wolffgramm’s animal model (Wolffgramm, 2004). Patients will most likely experience intrusions of an activated addiction memory as craving for the specific drug of abuse. In clinical studies, the reprocessing of these pathogenic craving memories within EMDR therapy improved the clinical course of patients with addiction memories (Hase et al., 2008; Abel and O’Brien, 2010).

(B) Patients with pain disorders. Phantom limb pain can be understood as the somatosensory intrusion of a pathogenic “pain memory.” One can assume that this memory is mainly based on the painful experiences before the limb was lost. Recent research showed that the prevalence of phantom limb pain after amputation of a limb or parts of it can be minimized by blocking nervous transmission for a prolonged period of time post-amputation, probably preventing the formation of pain memory (Borghi et al., 2010, 2014).

Reprocessing of pain memory should lead to symptom reduction. In three case series with a total of 30 phantom limb pain patients which were treated with EMDR therapy, 50% lost their pain completely (Schneider et al., 2005; Wilensky, 2006; de Roos et al., 2010). Additionally, Gerhardt et al. (2016) reported in a pilot study that patients with stressful memories and chronic back pain benefitted significantly from EMDR therapy, with 50% of patients losing their back pain completely.

(C) Patients with affective disorders. The importance of implicit memory in the pathogenesis of depression was already described by Barry et al. (2006). Recent studies link certain types of depression to stressful life events (Kendler et al., 2003). Until now, this was mainly considered a risk factor or a contributing factor for depression, but the concept of pathogenic memories offers another point of view. Since treatment options for recurrent depressive disorder patients and those with chronic depression are limited, further research investigating the role of depressive episode-triggering memories as well as EMDR therapy for the treatment of depressive disorders shows promise to improve the treatment of depression (Hofmann et al., 2014; Hase et al., 2015) and bipolar affective disorder (Landin-Romero et al., 2013; Novo et al., 2014).

### Summarizing on the AIP Model and Pathogenic Memories

The concept of pathogenic memories as the basis of mental and psychosomatic disorders can be easily integrated in the AIP model. The term “pathogenic memory” describes accurately the dysfunctionally stored memory as described by Shapiro in the AIP model. This opens up a new understanding of pathogenesis and therapeutic change in mental disorders far beyond PTSD. PTSD may be the prototypical disorder based in disruption of memory processing, but not the only one. These ideas could explain the development and progress of depression, the formation of pain memory leading to phantom limb pain, the role of addiction memory in addictive disorders, the deviational offender phantasies based on memories of abuse, the revenge phantasies of soldiers stemming from the battlefield memories and many more. On the other hand, EMDR therapy provides us not only with techniques to detect pathogenic memories but also with elaborated treatment plans (protocols), procedures, and techniques for a variety of mental disorders and has convincing evidence in the treatment of PTSD. This is a great advantage to Centonze’s appeal to remove pathogenic memories but lacking the tools to achieve this goal. Many studies on memory reprocessing in EMDR therapy with different disorders gave evidence on this AIP informed approach. It seems possible to target pathogenic memories and reprocess them, thus leading to transmutation, contributing to mental and physical equilibrium, and leading to long-lasting change.

## Discussion

There is a growing body of research showing that memories can contribute to pathology in many mental disorders. Research proposes to extend the range of disorders that are linked with pathogenic memories beyond PTSD and other trauma-based disorders. This is in line with the EMDR literature, where the AIP model of EMDR has predicted that PTSD is not the only memory-based disorder and has linked many other disorders to “dysfunctionally stored memories.”

One of the drawbacks of the AIP model is that it is difficult to determine what “dysfunctionally stored” means on a neurobiological level, which limits the scope of the AIP model. However, one could replace this term with the term “pathogenic” to define memories as causing symptoms without precisely needing to know their neurobiological details. In this way, more patients could benefit from a memory-related diagnosis and an adequate treatment. Meanwhile, research on memory pathology and its neurobiological underpinnings, as well as research on the clinical application of this knowledge could be supported by clear-cut research questions. This research direction also offers the possibility to move toward a diagnostic group of (mainly) “memory-based disorders” that are not exclusively focused on trauma-related events. This may lead to a broader application of well-researched EMDR protocols and procedures offering more help to patients who experience limited success undergoing psychotherapy as usual.

## Author Contributions

MH and AH laid the theme out and wrote the manuscript; UB contributed to the manuscript; and LO and PL assisted in the literature search.

## Conflict of Interest Statement

MH, PL, and AH are offering education in EMDR therapy to licensed psychotherapists. The other authors declare that the research was conducted in the absence of any commercial or financial relationships that could be construed as a potential conflict of interest. The reviewer CC declared a shared affiliation, though no other collaboration, with one of the authors, LO, to the handling Editor.
